# HIF-1α integrates lipogenic FASN and glycolytic GLUT3 to overcome intratumor oxidative and hypoxic stress for colorectal cancer metastasis

**DOI:** 10.1038/s41388-026-03738-4

**Published:** 2026-06-03

**Authors:** Yunyi Wang, Yuanyi Wei, Christopher Bailey, Gong Peng, Peng Zhang, Kunrong Cheng, Jean-Pierre Raufman, Yin Wang, Yan Liu

**Affiliations:** 1 Division of Immunotherapy, Institute of Human Virology, University of Maryland School of Medicine, Baltimore, MD USA; 2 Department of Cell Biology, China Medical University, Liaoning, China; 3 Institute of Translational Medicine, The First Hospital of Jilin University, Changchun, Jilin China; 4 Beijing Pediatric Research Institute, Beijing Children’s Hospital, Capital Medical University, National Cancer for Children’s Health, Beijing, China; 5 Division of Gastroenterology & Hepatology, University of Maryland School of Medicine, Baltimore, MD USA; 6 Department of Surgery and Comprehensive Cancer Center, University of Maryland School of Medicine, Baltimore, MD USA

**Keywords:** Targeted therapies, Stress signalling

## Abstract

Colorectal carcinoma (CRC) remains a leading cause of cancer mortality, largely due to metastasis. Solid tumors, including CRC, must adapt to intratumoral hypoxia and oxidative stress, but the tumor-cell programs that couple these pressures to metastatic competence remain unclear. Across human CRC cohorts and cell lines, HIF-1α was coordinately upregulated and co-expressed with the metabolic effectors GLUT3 and fatty-acid synthase (FASN), most prominently in metastatic lesions. Using HIF-1α (HRE), SREBP1 (SRE), and NRF2 (ARE) transcriptional reporters, we identified HRE-high and SRE-high CRC subpopulations with enhanced clonogenicity and invasion that drove accelerated tumor growth and increased lung metastatic burden across multiple CRC models. Mechanistically, IGF1 and insulin signaling through IGF1R and AKT-mTOR increased HIF-1α and induced FASN and GLUT3, enabling lipogenic, glycolytic, and antioxidant programs to withstand hypoxic and oxidative stress. HIF-1α engaged an HRE-containing proximal region of the human FASN promoter independently of SREBP1. Stress assays revealed functional specialization: FASN promoted NRF2-associated antioxidant capacity and resistance to oxidative injury, whereas GLUT3 preferentially supported hypoxia tolerance. In vivo, lipid nanoparticle–encapsulated echinomycin rapidly suppressed HRE, SRE, and ARE activity, reduced peri-hypoxic induction of FASN and GLUT3, inhibited tumor growth, and eliminated lung metastasis. These findings define a growth factor–responsive, HIF-1α-centered stress-adaptive state and highlight HIF-1α transcriptional activity as a therapeutic target in metastatic CRC.

## Introduction

Colorectal carcinoma (CRC) remains a major cause of cancer mortality worldwide, and metastatic dissemination is the principal driver of CRC-related deaths [1, 2]. Despite improvements in screening and systemic therapy, many patients present with already metastatic colon cancer or develop extra-colonic lesions after what was thought to be a curative resection [3, 4]. Responses of metastatic lesions to targeted therapies and immunotherapy are heterogeneous, and durable benefit is limited to defined molecular subsets, emphasizing the need to identify additional tumor intrinsic programs that enable progression and metastatic outgrowth [5–7].

A central pressure shaping tumor evolution is the microenvironment. Spatially heterogeneous oxygen and nutrient availability, driven by rapid tumor growth, creates cycles of hypoxia and oxidative stress that select for tumor cells with enhanced fitness and malignancy under stress, promoting local expansion, invasion, and metastasis [8]. Hypoxia inducible factor 1 alpha (HIF-1α) is a key regulator of these adaptations through transcriptional programs that remodel glycolysis, angiogenesis, survival pathways, and microenvironmental interactions [8]. Importantly, HIF-1α output is not determined solely by HIF1A RNA expression because HIF-1α transcriptional activity is dynamically regulated by oxygen tension, redox state, and growth factor signaling. Therefore, defining how functional HIF-1α activity relates to metastatic traits in solid tumors, including CRC remains a critical knowledge gap.

Systemic endocrine cues can further shape tumor metabolism. Insulin and insulin-like growth factor (IGF) signaling activate PI3K-AKT-mTOR pathways that couple growth signals to anabolic metabolism and survival [9–13]. Epidemiologic studies support an association between diabetes and increased CRC incidence and adverse outcomes, consistent with the concept that altered glucose metabolism and growth factor signaling can influence tumor progression, although the underlying mechanisms remain incompletely defined [14–16]. In CRC, elevated HIF-1α has been associated with poor prognosis and recurrence in several cohorts, whereas the contribution of HIF-2α appears less consistent, highlighting the need for mechanistic resolution of HIF-1α-driven programs linked to aggressive disease [17–20].

Metastatic fitness requires coordinated metabolic rewiring and stress tolerance. While HIF-1α-driven glycolysis is well established, de novo lipogenesis supports proliferation by supplying membrane components and signaling lipids and can contribute to cellular stress resistance [21–23]. Fatty acid synthase (FASN) is frequently elevated in CRC and correlates with advanced disease and poor outcome, and inhibition of FASN suppresses malignant phenotypes in preclinical models [24–26]. Sterol regulatory element binding protein 1 (SREBP1) is a canonical transcriptional regulator of lipogenic genes and is activated downstream of the IGF1 and insulin AKT-mTORC1 axis [27–30]. In parallel, enhanced glucose acquisition can support tumor survival under stress, and GLUT3-dependent adaptation has been implicated in CRC progression [31]. However, how growth factor signaling intersects with functional HIF-1α activity to coordinate lipogenesis, glucose utilization, and oxidative stress control within defined CRC subpopulations, and how this integration contributes to metastasis, remains unclear.

Here, we address this problem using transcriptional activity reporters to resolve pathway states at the single-cell level. We demonstrate that IGF1 and insulin, through their respective receptors and downstream AKT mTOR, promote HIF-1α accumulation and activity. HIF-1α induces FASN and GLUT3 and engages SREBP1 and NRF2, thereby allowing CRC cells to overcome intratumoral oxidative stress and hypoxia and metastasize to the lungs. Finally, leveraging strategies previously shown to suppress hypoxia-driven malignant programs [32–35], we found that targeting HIF-1α transcriptional activity with LNP echinomycin disrupts stress-adapted CRC metastatic traits and lung metastasis.

## Materials and methods

### Mice, cells, and reagents

#### Mice

Nod.Scid.Il2rg (NSG) and BALB/c mice (6–8 weeks old) were purchased from The Jackson Laboratory and used for CRC tumor inoculation and treatment studies. All experiments used mycoplasma-free cells and were performed in accordance with the NIH Guide for the Care and Use of Laboratory Animals. Animal procedures were approved by the Institutional Animal Care and Use Committee at the University of Maryland.

#### Cells

Human colorectal cancer cell lines (HCT116, HT29, Caco-2, LoVo, SW620, SW480) and the mouse colorectal cancer cell line CT26 were obtained from ATCC. Cells were maintained in RPMI-1640 supplemented with 10% fetal bovine serum (FBS), 2 mM glutamine, and penicillin/streptomycin (100 μ/mL each) at 37 °C with 5% CO₂. Cell lines were routinely tested for mycoplasma contamination and used at low passage after thawing.

#### Antibodies and reagents

Anti-HIF-1α (GTX127309, GeneTex); Cell Signaling antibodies: phospho-AKT (Ser473, #4060) and AKT (#9272), phospho-ERK1/2 (Thr202/Tyr204, #9101) and ERK (#9102), phospho-IGF1R β (Tyr980, #4568) and IGF1R β (#9750), phospho-GSK3α/β (Ser21/Ser9, #9331), cleaved Caspase-3 (#9661), FASN (#3189); Santa Cruz antibodies: mouse FASN (G-11, sc-48357), Ki-67/MIB-1 (sc-101861), EPAS-1/HIF2α (190b, sc-13596), and GAPDH (FL-335, sc-25778); SREBP1 (557036, BD Pharmingen), GLUT3 (NBP1-89762, Novus Biologicals), NRF2 (NBP1-32822, Novus Biologicals), and β-actin (AC-74, Sigma) were used. Additional reagents included BODIPY 493/503, BODIPY FL C16, MitoTracker Green FM, and DCFDA-Green (Thermo Fisher); 2-NBDG (Sigma); PD0325901 (Selleckchem); rapamycin, LY29004, and PD98059 (Cayman Chemical).

#### Plasmids and lentiviruses

Full-length HIF-1α cDNA and mature, active SREBP1α cDNA were cloned into pcDNA expression vectors. A triple-mutant HIF-1α (P402A/P564A/N802A; HIF-1α-PPN) was generated by site-directed mutagenesis. pcDNA3.1-FASN (Addgene #107138) was obtained from Addgene. HIF-1α knockout in HCT116 cells was generated using CRISPR/Cas9 as described previously [36]. Lentiviral shRNAs targeting HIF-1α, GLUT1, GLUT3, FASN, and IGF1R, along with a scrambled control shRNA, and transcriptional reporter lentiviruses for HIF-1α (HRE), SREBP1 (SRE), and NRF2 (ARE) (GFP-Puro or RFP-BSD), were purchased from LipExoGen. For each target gene, two independent shRNA lentiviruses were pooled at equal titer to generate polyclonal stable knockdown populations.

### Promoter reporter and chromatin immunoprecipitation (ChIP) assays and EMSA

#### Promoter reporter construction

The proximal promoter region of human FASN was PCR-amplified from genomic DNA isolated from healthy human PBMCs using the following primers: forward, 5′-CGGTGTGGCCACGGGGTAGTC-3′; reverse, 5′-AGGCTGAAGCGCGGCGGAGAG-3′. The PCR product was cloned into a lentiviral promoter reporter vector (LipExoGen) upstream of an EGFP reporter cassette and a blasticidin resistance marker.

### ChIP-qPCR

HRE-high HCT116 cells were serum-starved for 8 h, stimulated with IGF1 (20 ng/mL) for 2 h, and crosslinked with 1% formaldehyde. Cells were lysed, and chromatin was sheared by sonication. Sonicated lysates were diluted 1:10 and subjected to immunoprecipitation with an anti-HIF-1α antibody; normal IgG was processed in parallel as a negative control. ChIP was performed using a ChIP Assay Kit (17-295, Millipore) according to the manufacturer’s instructions. Following reversal of crosslinks and DNA purification, enrichment at target loci was assessed by PCR using the following primers: P1-F1 (5’-CGGTGTGGCCACGGGGTAGTC-3’) and P1-R1 (5’-GGGCTGGGACTGAGGAGCG-3’); P2-F2 (5’-AGGCGCGTTCCCGCGCAGG-3’) and P2-R2 (5’-AGGCTGAAGCGCGGCGGAGAG-3’).

### EMSA

HRE-high HCT116 cells were serum-starved for 8 h and treated with IGF1 (20 ng/mL) for 2 h. Nuclear extracts were prepared, and 10 µg nuclear protein was used per binding reaction. EMSA was performed using the Pierce LightShift Chemiluminescent EMSA Kit (20148, Thermo Scientific) to detect HIF-1α binding to HRE-containing double-stranded DNA probes biotin-labeled at the 3′ end. Complexes were resolved by native gel electrophoresis, transferred to membrane, and detected using streptavidin-conjugated HRP and a chemiluminescent substrate. The DNA probes used were: P1: CACACGTGGCCCCGGCGGACACGGGG; P2: CAGCCCATGTGGCGTGGCCGCCCG.

### Treatment and transduction

#### Mice treatments

NSG and BALB/c mice (both sex, 8–10 weeks, 5–6/group) were inoculated with 1 × 10⁶ CRC cells (reporter or shRNA) in the inguinal area. Tumors were measured twice weekly until reaching protocol-required size. Mice received intravenous doses of Lipo-EM (250 μg/kg) in liposomal formulation or LNP-EM (250 μg/kg) in lipid nanoparticle formation or PD0325901 (2 mg/kg) in liposomal formation, alone or combined with 250 μg/kg Lipo-EM, every 3 days for 3 doses. Control mice received an empty liposome or LNP. The liposomal formations were prepared primarily as we described before [33]. Animals were randomized into groups. All animal experiments have been performed at least twice. All animal studies were blinded and conducted under the guidelines of the Institutional Animal Care and Use Committee of the University of Maryland.

#### In vivo hypoxia probe assay

Mice were administered hypoxyprobe (pimonidazole) 90 min before euthanasia, following the manufacturer's instructions. Tumors were sectioned and stained for hypoxyprobe, HIF-1α, NRF2, SREBP1, Ki67, cleaved Caspase 3, FASN, GLUT3, and 4′,6-diamidino-2-phenylindole (DAPI).

#### Cell treatments

CRC cell lines or lentivirally transduced stable cells were serum-starved for 8 h before being treated with growth factors or serum. CoCl₂ treatment (250 µg/mL) was applied to overnight-passaged cells at approximately 80% confluence in 10% FBS medium without serum starvation for 16 h. Similarly, freshly diluted hydrogen peroxide (H₂O₂) at 500 µM was added for 30 min prior to performing colony formation assays and MTT assays. For fluorescence observation, cells were labeled with ROS-665 dye (1 µM) for 30 min, then washed with PBS and fixed in 4% paraformaldehyde (PFA). Bodipy FL C16, Mito-Tracker Green FM, and ROS DCFDA were each added at 1 µM for 30 min following an 8-h serum starvation, with or without a subsequent 2-h treatment with 20 ng/mL IGF1. The uptake of the fluorescent glucose analogue NB2DG was assessed at 60 µM for 1 h following an 8-h glucose-free starvation in 2.5% FBS DMEM. For stress condition assays, cells were cultured in 10% FBS Dulbecco’s modified Eagle’s medium (DMEM) medium under glucose deprivation (medium without glucose), glycolysis inhibition (medium with 10 mM 2-DG), or acidosis (medium adjusted with lactic acid to maintain pH 6) for 24 h before performing the MTT assay to evaluate the resulting cellular stress.

#### Human colon cancer tissue

Human CRC tissue was obtained from the surgical resection of a 76-year-old woman with synchronous cecal adenocarcinoma (poorly differentiated, grade 3; pathologic stage pT4a pN1a pMX) and processed according to institutional protocols for downstream functional and reporter-based analyzes. The study was approved by the University of Maryland School of Medicine Institutional Review Board, and written informed consent was obtained from the subject; samples were de-identified prior to analysis.

#### Lentiviral transduction

CRC cell lines (1–3 × 10⁵ cells/well in a 12-well plate) were transduced with 25–50 μL of reporter or shRNA lentiviruses in the presence of 6 μg/mL polybrene. After 3 days, the cells were transferred to a 6-well plate and cultured in medium with 2 µg/mL puromycin, refreshed every 2 days until colonies formed. Drug-resistant polyclonal stable cells were used for experiments.

### Western-blot

After treatments, CRC cells were lysed directly in the culture plate using 2X SDS loading buffer with 2 mM DTT, phosphatase inhibitor cocktail (1×, Roche 4906845001), and protein inhibitor cocktail (1×, Sigma). Lysates were sonicated, and ~50–100 µg of protein (determined by bicinchoninic acid (BCA) Protein Assay, Thermo Fisher 23225) was resolved on a 4–12% sodium dodecyl sulfate-polyacrylamide gel electrophoresis (SDS-PAGE) gradient gel. Proteins were transferred to PVDF membranes (Millipore, ISEQ00010), blocked with 5% bovine serum albumin (BSA), and incubated overnight at 4 °C with primary antibodies. After washing, membranes were incubated with HRP-linked secondary antibodies for chemiluminescence detection.

### Histology and immunohistochemistry

Fresh tissues were fixed in 10% neutral formalin, paraffin-embedded, sectioned at 4 μm, and mounted on slides. Sections were deparaffinized, hydrated, and stained with hematoxylin and eosin (H&E). Tumor tissues from mice were immediately frozen after intraperitoneal injection of a hypoxyprobe for 90 min. Frozen sections (5 μm) were used for fluorescence staining. CRC tissue microarrays (US BioMax, CO702d) were antigen-retrieved and processed as previously described [35], then co-stained with rabbit anti-HIF-1α (GTX127309) and mouse anti-FASN (Santa Cruz) overnight at 4 °C. Sections were washed with PBS + 0.01% Tween 20 (PBST), incubated with secondary antibodies for 2 h at room temperature, washed again, and mounted in antifade medium with DAPI. Images were captured using an Olympus IX73 fluorescence microscope.

### RNA extraction and real-time PCR

Total RNA was isolated from tissues/cells using Trizol reagent (Invitrogen, 15596026), followed by reverse transcription with SuperScript III for RT-qPCR (Thermo Fisher). qPCR was conducted with SYBR Green (Roche, 04913914001) on a Step One Plus system (Applied Biosystems), with GAPDH as the reference gene.

### Statistical analysis

Statistical analysis was performed using SPSS 21.0. Quantitative data are presented as mean ± SD unless noted otherwise. Statistical significance was assessed with unpaired 2-tailed Student’s *t*-test, 2-way ANOVA, log-rank test, or Wilcoxon signed-rank nonparametric test, as appropriate. Survival comparisons used the log-rank test. Significance levels were denoted as follows: ^*^p < 0.05, ^**^p < 0.01, ^***^p < 0.001, ^****^p < 0.0001.

## Results

### HIF-1α and FASN co-expression in metastatic CRC and identification of HIF-1α and SREBP1 activity-defined subpopulations

**Fig. 1:**
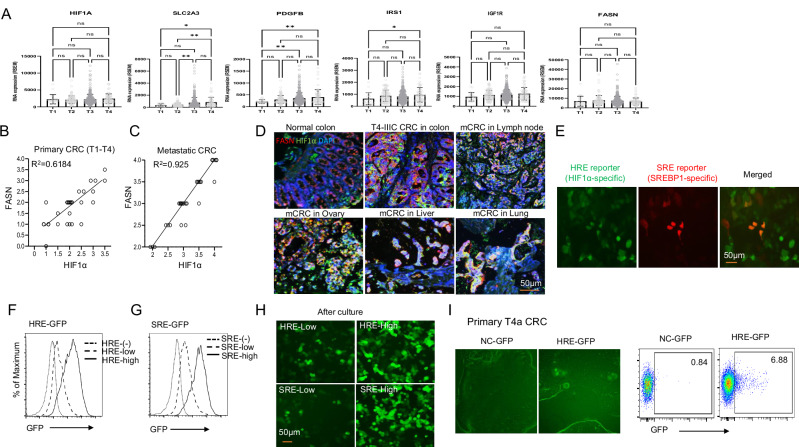
Co-expression of HIF1α and FASN in metastatic CRCs. **A** TCGA colorectal cancer RNA-seq (RNAseqV2 normalized RSEM; *n* = 589) analysis showing RNA expression of *HIF1A* and related genes/targets (*SLC2A3, PDGFB*), IGF1R pathway components (*IRS1, IGF1R*), and *FASN* across tumor T stages (T1-T4); significance as indicated on the plot. ^*^P < 0.05; ^**^P < 0.01; ^***^P < 0.001; ^****^P < 0.0001. Correlation between HIF-1α and FASN protein levels in CRC tissue microarrays based on immunofluorescence intensity scoring (scale 1-4) in primary CRC (T1-T4) (**B**) and metastatic CRC (**C**); linear regression with *R*² values shown. **D** Representative merged immunofluorescence images of normal colon, primary CRC (example shown: T4-IIIC), and metastatic CRC lesions (lymph node, ovary, liver, lung) stained for FASN and HIF-1α with DAPI nuclear counterstain. **E** Parental HCT116 cells co-transduced with HIF-1α-specific hypoxia response element (HRE) reporter and SREBP1-specific sterol regulatory element (SRE) reporter lentiviruses; representative fluorescence images after antibiotic selection. Representative flow-cytometry histograms showing reporter fluorescence distributions and gates used to define reporter-negative, -low, and -high populations for HRE (**F**) and SRE (**G**). **H** Representative fluorescence images of sorted HRE-low/high and SRE-low/high populations after culture, demonstrating maintenance of differential reporter activity. **I** Primary T4a CRC patient-derived cells (passage 1) were cultured in RPMI 1640 supplemented with 10% FBS and transduced with either negative-control GFP or HRE-GFP lentivirus for 3 days, followed by selection with puromycin (1.5 µg/mL) for 7 days. Representative fluorescence images (left) and flow-cytometry quantification of GFP^+^ cells (right; percentages shown) are presented. The data are representative of 2 independent experiments.

To assess how HIF1A and pathway-associated metabolic genes change during CRC progression, we analyzed RNA-seq data from 589 colorectal adenocarcinoma samples in The Cancer Genome Atlas (TCGA) using RNAseqV2 normalized RSEM values with matched clinical annotation. Consistent with a stress-adaptive program emerging in advanced disease, expression of SLC2A3 (GLUT3) and PDGFB was higher in T3 and T4 tumors compared with T1 tumors, and IRS1 also increased with stage (Fig. 1A). In contrast, HIF1A and FASN RNA levels showed only modest differences across T stages, highlighting that RNA abundance of HIF1A may not fully capture functional HIF-1α pathway activation within heterogeneous tumors (Fig. 1A). We next evaluated HIF-1α and FASN at the protein level in patient tissues. Immunofluorescence staining of CRC tissue microarrays spanning primary tumors (T1 to T4) and metastatic lesions revealed a positive association between HIF-1α and FASN protein signals. Notably, the correlation was substantially stronger in CRC metastases than in primary CRC (Fig. 1B, C). Representative images demonstrate co-localization patterns in metastases from major CRC target organs, including lymph node, liver, and lung (Figs. 1D and S1). These observations suggest that coupling of HIF-1α and lipogenic programs is most prominent in clinically advanced disease. To functionally interrogate HIF-1α and lipogenic pathway activity at the single-cell level, we transduced HCT116 cells with lentiviral transcriptional reporters driven by a HIF-1α-specific hypoxia response element (HRE) and a SREBP1-responsive sterol regulatory element (SRE). Fluorescence imaging confirmed reporter expression in transduced populations and showed enrichment of HRE-high cells within the SRE-high compartment (Fig. 1E). Flow cytometry identified distinct reporter-defined subsets, enabling gating of HRE-negative/low/high and SRE-negative/low/high fractions (Figs. 1F, G and S2). Sorted HRE-high and SRE-high cells maintained elevated reporter activity after culture (Fig. 1H). Finally, to extend the reporter strategy to clinically relevant material, we infected primary T4a CRC patient-derived cells with HRE-GFP lentivirus and observed a measurable HRE-positive fraction compared with negative-control GFP lentivirus (Fig. 1I). Together, these data establish a patient-supported link between HIF-1α and FASN in metastatic CRC and provide a reporter-based platform to isolate and interrogate HIF-1α and SREBP1 activity-defined CRC subpopulations.

### HRE-high and SRE-high CRC cells display enhanced clonogenicity, tumor growth, and lung metastatic potential

**Fig. 2:**
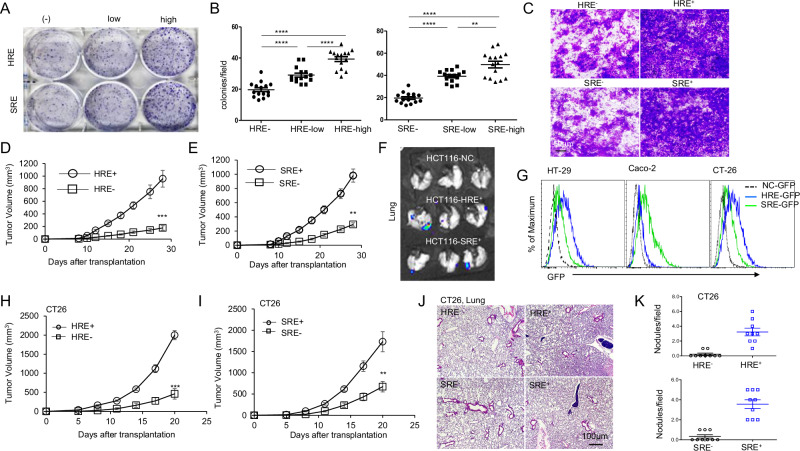
HRE-high and SRE-high CRC cells display enhanced clonogenicity, invasion, tumor growth, and lung metastasis. **A**, **B** Colony formation of sorted HCT116 reporter-defined populations (HRE-negative/low/high or SRE-negative/low/high, as indicated). Cells (1 × 10⁴ per well) were plated in 6-well plates, cultured for 7 days, stained with crystal violet, and colonies quantified from 15 fields per condition. Data were analyzed by 1-way ANOVA with Sidak’s multiple-comparison test. **C** Transwell invasion assay using the indicated HCT116 populations. Cells (1 × 10⁵ per insert; 6.8 μm pores) were cultured for 3 days and stained with crystal violet. **D**–**F** In vivo tumor growth and lung metastasis of reporter-defined HCT116 subpopulations. HRE-positive, SRE-positive, and negative-control (NC) cells (1 × 10⁶ cells per mouse) were injected into the inguinal region of mice (*n* = 5 per group). Tumor growth kinetics are shown in **D**, **E**, and lung metastasis was assessed by luciferase imaging on day 28 post-implantation (**F**). **G** Reporter activity profiling across additional CRC cell lines transduced with HRE, SRE, or NC lentiviral reporters and analyzed by flow cytometry. **H**–**K** Validation in an immunocompetent model. CT26 HRE-positive or SRE-positive cells (1 × 10⁶) were injected into the inguinal area of BALB/c mice. Tumor growth kinetics (**H**, **I**), lung metastasis (**J**), and lung tumor nodule quantification from FFPE sections (10 fields per mouse; K) are shown. *n* = 5 mice per group. Data are presented as mean ± SEM for each group and were analyzed by Student’s *t* test (**K**). The data are representative of 2 independent experiments. Mean tumor volumes ± SEM are plotted on the *y* axis for each group (*n* = 5 per group) and were analyzed by two-way ANOVA (**D**, **E**, **H**, **I**).^*^P < 0.05; ^**^P < 0.01; ^****^P < 0.0001.

To test whether pathway activity marks functionally aggressive CRC subpopulations, we compared reporter-defined fractions in vitro and in vivo. In colony formation assays, HRE-high and SRE-high HCT116 cells formed substantially more colonies than the corresponding reporter-negative and reporter-low fractions (Fig. 2A, B). Consistent with an invasive phenotype, reporter-positive populations also exhibited increased transwell migration compared with reporter-negative controls (Fig. 2C). We next assessed tumorigenic and metastatic capacity in vivo. Following implantation into the inguinal region of immunodeficient NSG mice, HRE-positive and SRE-positive HCT116 cells generated tumors with accelerated growth kinetics relative to their reporter-negative counterparts (Fig. 2D, E). Bioluminescence imaging of lungs revealed increased metastatic dissemination from mice bearing HRE-positive or SRE-positive tumors (Fig. 2F). These data indicate that elevated HIF-1α or SREBP1 transcriptional activity identifies CRC cell fractions with enhanced tumor growth and lung metastatic potential. To determine whether reporter-positive subsets are a general feature of CRC, we introduced HRE and SRE reporters into additional CRC lines. Distinct reporter-positive fractions were detected in human HT-29 and Caco-2 CRC cell lines, as well as in the murine CT26 CRC cell line (Fig. 2G), supporting the presence of conserved pathway activity states across CRC models. Finally, to evaluate these states in an immunocompetent context, we implanted CT26 reporter-defined populations into BALB/c mice. Both HRE-positive and SRE-positive CT26 cells exhibited markedly faster tumor growth than reporter-negative cells (Fig. 2H, I). Lung metastasis was confirmed by histology (Fig. 2J) and quantified as increased lung tumor nodule formation in mice implanted with HRE-positive or SRE-positive CT26 cells (Fig. 2K). Together, these results establish that HRE and SRE reporter activity prospectively enriches for CRC subpopulations with increased proliferative and metastatic capacity across multiple cell line backgrounds and in both immunodeficient and immunocompetent models.

### HRE-positive CRC cells display an IGF1/insulin responsive HIF-1α program that induces FASN and GLUT3

**Fig. 3:**
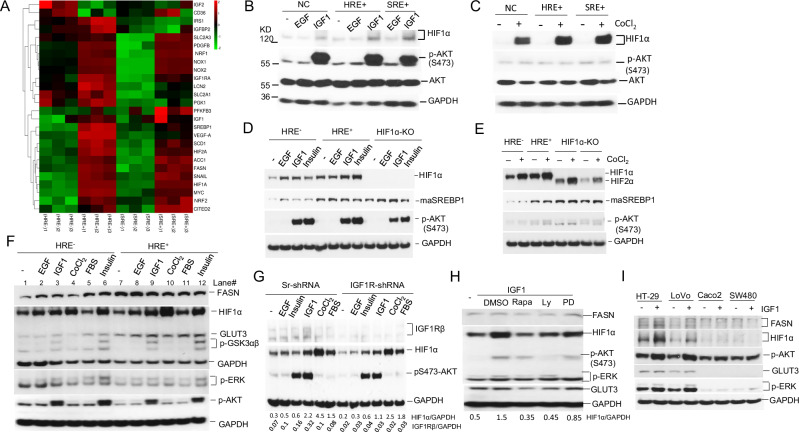
HIF-1α, FASN, and GLUT3 are enriched in HRE+ cells and are induced by growth factors primarily through IGF1R and AKT-mTOR signaling. **A** Heatmap of RT-qPCR showing relative RNA levels of metabolic, oxidative-stress, and oncogenic pathway genes in HRE+ and SRE + HCT116 cells compared with negative-control cells. **B** Immunoblot analysis of HIF-1α accumulation and AKT activation (p-AKT S473) in negative-control (NC), HRE+, and SRE+ cells following EGF or IGF1 stimulation (IGF1, 20 ng/mL) after 8 h serum starvation. **C** Immunoblot analysis of HIF-1α accumulation in NC, HRE+, and SRE+ cells following CoCl2 stimulation (250 μg/mL). Immunoblot analysis of SREBP1 maturation (maSREBP1) in HRE-, HRE+, and HIF-1α-knockout (HIF-1α-KO) HCT116 cells after stimulation with growth factors (**D**) or CoCl2 (**E**), with p-AKT (S473) as a pathway readout. **F** Immunoblot showing that IGF1 and insulin elevate HIF-1α with concomitant induction of FASN and GLUT3 and activation of downstream signaling (p-GSK3αβ, p-ERK, p-AKT) in HRE^−^ versus HRE^+^ cells under the indicated conditions. Lanes are numbered; HRE^−^ (lanes 1–6) and HRE^+^ (lanes 7–12) are shown in parallel and compared as lane-matched pairs: 1/7 (-), 2/8 (EGF), 3/9 (IGF1), 4/10 (CoCl2), 5/11 (FBS), and 6/12 (insulin). Where densitometry is reported, quantification used these matched pairs and was normalized to GAPDH. **G** Immunoblot showing that IGF1-induced HIF-1α accumulation is mediated by IGF1R signaling. Cells expressing scramble shRNA or IGF1R shRNA were stimulated as indicated; HIF-1α/GAPDH ratios are shown below. **H** Pathway mapping of IGF1-induced signaling. Cells were treated with vehicle (DMSO) or inhibitors (Rapa, Ly, PD) prior to IGF1 stimulation; immunoblots show effects on HIF-1α and downstream targets (FASN, GLUT3) and pathway readouts (p-AKT, p-ERK). **I** Validation in additional CRC cell lines (HT-29, LoVo, Caco-2, SW480) stimulated with IGF1 (20 ng/mL), showing induction of HIF-1α, FASN, and GLUT3 with pathway activation. The data are representative of 3 independent experiments.

To define the molecular programs associated with reporter-defined states, we profiled HRE-positive and SRE-positive HCT116 cells by RT-qPCR. Heatmap analysis of RNA levels showed that both HRE-positive and SRE-positive populations were enriched for transcripts linked to metabolic adaptation and malignant progression, including genes involved in glucose transport and glycolysis, lipogenesis, growth-factor signaling, oxidative-stress responses, angiogenesis, and EMT-related programs, relative to reporter-negative controls (Fig. 3A). We next examined how these subpopulations respond to growth-factor signaling and hypoxia-mimicking stress. Upon stimulation with IGF1, HRE-positive and SRE-positive cells exhibited robust AKT activation and increased HIF-1α accumulation compared with negative-control (NC) reporter cells, whereas EGF produced weaker effects under these conditions (Fig. 3B). CoCl₂ induced HIF-1α in all groups, but HRE-positive and SRE-positive cells maintained higher HIF-1α levels than NC cells (Fig. 3C), consistent with a pathway-primed state. Together, these results indicate substantial overlap between HRE-positive and SRE-positive subsets. In parallel, HRE-positive cells displayed elevated basal levels of mature SREBP1, and growth-factor stimulation did not further increase maSREBP1 compared with HRE-negative cells (Fig. 3D). In contrast, in HIF-1α knockout cells, maSREBP1 was increased and remained responsive to EGF, IGF1, and insulin, resembling HRE-negative cells (Fig. 3D). Under CoCl₂ exposure, maSREBP1 was not induced in HIF-1α knockout cells (Fig. 3E). Together, these findings suggest that HIF-1α status did not influence SREBP1 maturation in CRC cells. Consistent with a downstream metabolic program, elevated HIF-1α in HRE-positive cells was accompanied by increased expression of FASN and GLUT3 following growth-factor stimulation and CoCl₂ treatment, compared with HRE-negative cells (Fig. 3F). Mechanistically, IGF1R contributed to IGF1-induced HIF-1α accumulation, as IGF1R knockdown reduced, but did not abolish, HIF-1α induction and downstream signaling (Fig. 3G). Pharmacologic mapping further supported an IGF1R–AKT–mTOR route, as inhibition of mTOR with rapamycin markedly suppressed IGF1-driven HIF-1α accumulation and reduced induction of GLUT3 and FASN (Fig. 3H). Finally, IGF1-dependent induction of HIF-1α and FASN with concordant pathway activation was observed in HT-29 and LoVo cells, supporting extension of this response beyond HCT116 cells (Fig. 3I). Collectively, these data position IGF1/insulin signaling as an upstream driver of a HIF-1α-high transcriptional state that promotes coordinated induction of lipogenic and glucose-uptake programs in CRC cells.

### HIF-1α drives CRC tumor growth and lung metastasis through induction of FASN and GLUT3

**Fig. 4:**
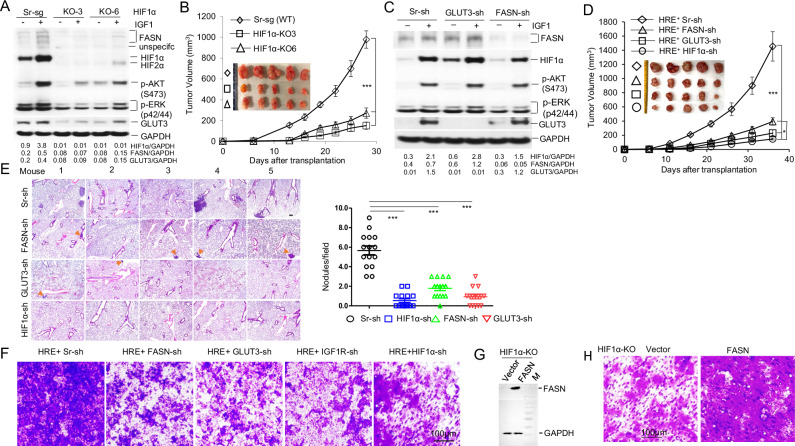
HIF-1α promotes CRC tumor growth and lung metastasis by upregulating FASN and GLUT3. **A** Immunoblot analysis of the indicated proteins in parental HCT116 cells (Sr-sg, WT) and two independent HIF-1α-knockout clones (HIF-1α-KO3 and HIF-1α-KO6) under basal conditions or after IGF1 stimulation (±IGF1, as indicated). **B** In vivo tumor growth kinetics of Sr-sg (WT) and HIF-1α-KO HCT116 cells following inguinal implantation (1 × 10⁶ cells per mouse; *n* = 5 mice per group). Representative endpoint tumors are shown. **C** Immunoblot validation of knockdown in HRE+ HCT116 cells expressing scramble shRNA (Sr-sh) or shRNAs targeting GLUT3 or FASN, with or without IGF1 stimulation (±IGF1, as indicated). **D** Tumor growth kinetics of HRE+ HCT116 cells expressing Sr-sh, FASN-sh, GLUT3-sh, or HIF-1α-sh after inguinal implantation (1 × 10⁶ cells per mouse; *n* = 5 mice per group). Representative endpoint tumors are shown. **E** Lung metastasis assessment. Representative H&E images of lung sections from mice bearing HRE+ HCT116 tumors with the indicated knockdowns (mouse #1–5 shown). Lung tumor nodules were quantified from 10 fields per mouse using FFPE sections (right). Data were analyzed by 1-way ANOVA with Sidak’s multiple-comparison test. **F** Transwell invasion assays of HRE+ HCT116 cells expressing Sr-sh, FASN-sh, GLUT3-sh, IGF1R-sh, or HIF-1α-sh. Cells (1 × 10⁵ per well) were stained with crystal violet after 3 days. FASN rescue in HIF-1α-KO cells. Immunoblot confirming restoration of FASN expression (**G**) and representative transwell invasion images showing increased invasive capacity following FASN re-expression (**H**). Mean tumor volumes ± SEM are plotted on the *y* axis for each group (*n* = 5 per group) and were analyzed by two-way ANOVA (**B**, **D**). The data are representative of 2 independent experiments. ^***^P < 0.001.

To determine whether HIF-1α is required for malignant phenotypes in CRC, we generated two independent HIF-1α knockout (KO) HCT116 clones using CRISPR Cas9. Immunoblot analysis showed that loss of HIF-1α reduced expression of the HIF-1α associated metabolic targets FASN and GLUT3 under basal conditions and following IGF1 stimulation, while HIF2α was increased in a compensatory manner (Fig. 4A). In these HIF-1α -KO cells, IGF1 responsive signaling was altered, with reduced downstream pathway readouts compared with control cells (Fig. 4A). We next tested the requirement for HIF-1α in vivo. Compared with scramble sgRNA controls, both HIF-1α -KO clones formed substantially smaller tumors following implantation, demonstrating that HIF-1α is critical for efficient tumor outgrowth in this model (Fig. 4B). To examine downstream effectors within the HIF-1α high state, we knocked down candidate targets in HRE^+^ HCT116 cells. Immunoblotting confirmed effective knockdown of FASN or GLUT3 and preserved IGF1-induced AKT pathway activation (Fig. 4C), supporting that the observed phenotypes reflect disruption of these metabolic nodes rather than global blockade of IGF1 signaling. In vivo, knockdown of HIF-1α, FASN, or GLUT3 in HRE^+^ cells markedly reduced tumor growth compared with scramble shRNA controls (Fig. 4D). Consistent with impaired metastatic competence, histologic analysis of lungs demonstrated decreased metastatic lesions following knockdown of HIF-1α, FASN, or GLUT3 (Fig. 4E). In parallel, transwell assays showed that depletion of HIF-1α, IGF1R, FASN, or GLUT3 reduced invasive capacity of HRE^+^ cells (Fig. 4F), linking this pathway to invasion-associated traits. Finally, to test whether FASN functions as a key downstream effector of HIF-1α, we re-expressed FASN in HIF-1α-KO cells. Restoration of FASN protein was confirmed by immunoblotting (Fig. 4G), and FASN rescue increased invasive capacity compared with vector control (Fig. 4H). Together, these data establish that HIF-1α is required for CRC tumor growth and lung metastasis and identify FASN and GLUT3 as functionally important downstream metabolic effectors that mediate HIF-1α-driven malignant phenotypes.

### HIF-1α directly activates the FASN promoter and promotes lipogenic and redox-associated phenotypes in HRE-positive CRC cells

**Fig. 5:**
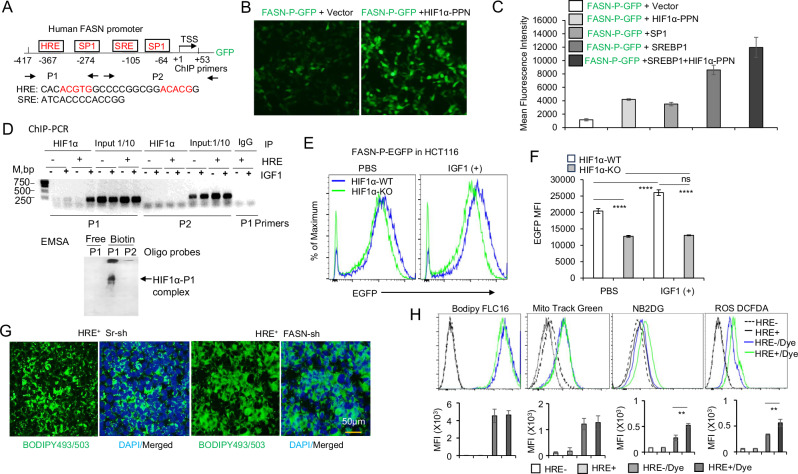
HIF-1α directly activates the human FASN promoter and enhances lipogenesis in HRE+ CRC cells. **A** Schematic of the proximal human FASN promoter (−417 to +53) showing the predicted transcription factor binding sites (HRE, SP1, SRE), transcription start site (TSS), and locations of the ChIP-qPCR primer sets (P1 and P2). **B**, **C** HEK293FT cells were co-transfected with the FASN promoter–EGFP reporter (FASN-P-GFP) together with the indicated expression vectors (Vector, stabilized HIF-1α mutant HIF-1α-PPN, SP1, active SREBP1, or SREBP1 plus HIF-1α-PPN). Representative fluorescence images are shown in (**B**), and promoter activity was quantified by flow cytometry as mean fluorescence intensity (MFI) in **C**. **D** ChIP-PCR demonstrates HIF-1α occupancy at the HRE-containing promoter region (P1) following serum starvation and IGF1 stimulation, with IgG as a negative IP control and input DNA included. EMSA using P1 and P2 oligonucleotide probes further supports formation of an HIF-1α-P1 DNA complex. **E**, **F** HIF-1α-WT (sgRNA control) and HIF-1α-KO HCT116 cells stably expressing the FASN promoter reporter were stimulated with IGF1 (or PBS vehicle), and EGFP reporter activity was assessed by flow cytometry. Representative histograms are shown in **E**, with summary EGFP MFI quantification in **F** (statistics as indicated in the panel). **G** Representative fluorescence images of HRE+ HCT116 cells expressing control shRNA (Sr-sh) or FASN shRNA (FASN-sh) stained with BODIPY 493/503 (green) to visualize neutral lipid droplets (punctate green droplets) and counterstained with DAPI (blue). Note the marked reduction of punctate intracellular BODIPY-positive lipid droplet signal in FASN-sh cells. **H** Following serum starvation, HRE- and HRE+ HCT116 cells were stimulated with IGF1 and analyzed for metabolic/oxidative phenotypes by flow cytometry, including fatty-acid uptake (BODIPY FL C16), mitochondrial mass (MitoTracker Green), glucose uptake (2-NBDG), and intracellular ROS (DCFDA). Representative histograms (top) and MFI quantification (bottom) are shown (statistics as indicated). The data are representative of 2 independent experiments. Data are presented as mean ± SEM for each group and were analyzed by Student’s *t* test (**C**, **F**, **H**). ^*^P < 0.05; ^**^P < 0.01; ^****^P < 0.0001.

Given the strong association between HIF-1α and FASN in metastatic CRC, we asked whether HIF-1α can directly regulate FASN transcription. Inspection of the proximal human FASN promoter identified a candidate HIF binding region containing a consensus HRE adjacent to SP1 and SRE motifs (Fig. 5A). To test transcriptional regulation, we generated a FASN promoter-driven EGFP reporter (FASN-P-GFP). In HEK293FT cells, expression of a stabilized HIF-1α mutant (HIF-1α PPN) increased FASN promoter reporter activity relative to vector control, and co-expression with lipogenic transcription factors further enhanced reporter output (Fig. 5B, C), indicating that the proximal promoter region is responsive to HIF-1α-dependent signaling. We next evaluated direct binding of endogenous HIF-1α to the FASN promoter. ChIP PCR demonstrated enrichment of HIF-1α on the HRE-containing promoter region in response to IGF1 stimulation, whereas IgG controls did not show enrichment (Fig. 5D). Consistent with specific DNA binding, EMSA using oligonucleotides spanning the HRE containing region supported formation of a HIF-1α DNA complex (Fig. 5D). To determine whether HIF-1α is required for FASN promoter activity in CRC cells, we introduced the FASN promoter EGFP reporter into HIF-1α wild type (sgRNA control) and HIF-1α-KO HCT116 cells. Reporter activity was significantly reduced in HIF-1α-KO cells under basal conditions and after IGF1 stimulation, demonstrating that HIF-1α is necessary for full FASN promoter activation in this context (Fig. 5E, F). Notably, this effect occurred despite increased mature SREBP1 observed in HIF-1α-KO cells (Fig. 3D), supporting a direct and SREBP independent contribution of HIF-1α to FASN transcriptional control. Functionally, HRE positive HCT116 cells exhibited increased neutral lipid droplets, as visualized by BODIPY staining, and this phenotype was attenuated by FASN knockdown (Fig. 5G), linking HIF-1α-dependent FASN induction to enhanced lipogenesis. Finally, flow cytometry based metabolic profiling indicated that HRE positive cells displayed increased glucose uptake and higher intracellular ROS signals relative to HRE negative cells, whereas fatty acid uptake and mitochondrial mass showed limited changes under these conditions (Fig. 5H). Together, these results identify HIF-1α as a direct activator of the human FASN promoter and support a model in which elevated HIF-1α activity promotes a lipogenic program in HRE positive CRC cells that is coupled to altered redox state and stress adaptation.

### HIF-1α coordinates antioxidant and stress-resistance programs, with FASN and GLUT3 providing distinct protection against oxidative and hypoxic stress

**Fig. 6:**
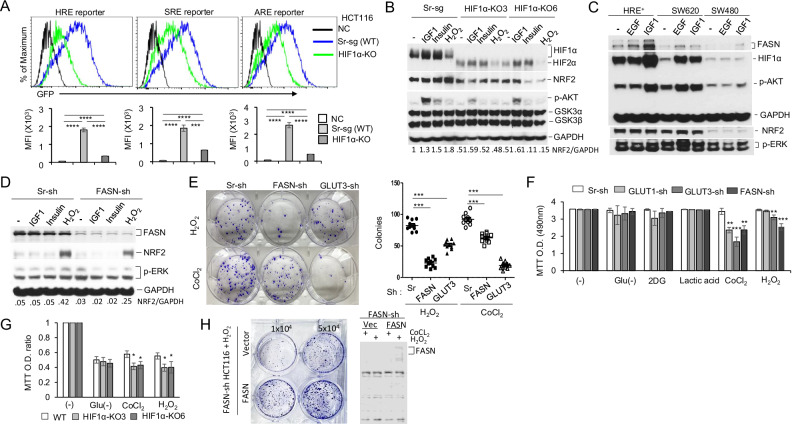
HIF-1α coordinates FASN- and GLUT3-dependent defense against oxidative stress and hypoxic injury. **A** HIF1α-WT (Sr-sg) and HIF1α-KO HCT116 cells were transduced with GFP-based TF reporter lentiviruses (HRE, SRE, or ARE). Reporter activity was measured by flow cytometry; representative GFP histograms (top) and quantification of GFP mean fluorescence intensity (MFI; bottom) are shown, with NC as the reporter-negative control. **B** Immunoblot analysis of NRF2 and indicated signaling proteins in Sr-sg (WT) and two independent HIF-1α-KO clones (KO3, KO6) following stimulation with IGF1, insulin, or H₂O₂ (H₂O₂: 500 μM, 30 min). **C** Immunoblot analysis of FASN, HIF-1α, NRF2, and pathway readouts in HRE+ HCT116 cells and the metastatic CRC line SW620 compared with SW480 under basal conditions or after growth-factor stimulation (EGF or IGF1, as indicated). **D** Immunoblot showing that FASN knockdown in HRE+ cells reduces basal and H₂O₂-induced NRF2 protein accumulation (NRF2/GAPDH ratios shown). **E** Colony growth assays under oxidative stress (H₂O₂) or hypoxic stress (CoCl₂). FASN-sh cells show increased sensitivity to oxidative stress, whereas GLUT3-sh cells show increased sensitivity to hypoxic stress; quantification is shown at right. Data were analyzed by 1-way ANOVA with Sidak’s multiple-comparison test. **F** MTT viability assays under the indicated stresses show that knockdown of FASN or GLUT3 has limited impact on viability under glucose deprivation (Glu(-)), glycolysis inhibition (2DG), or lactic acid exposure, but more strongly reduces viability under oxidative (H_2_O_2_) and hypoxia-mimicking (CoCl_2_) stress (conditions as indicated). **G** HIF-1α-KO HCT116 clones display increased sensitivity to hypoxia-mimicking (CoCl_2_) and oxidative (H_2_O_2_) stress compared with WT cells in the short-term MTT assay (MTT O.D. ratio); Glu(-) denotes glucose deprivation. **H** FASN rescue experiment: transient re-expression of FASN in FASN-sh HRE^+^ HCT116 cells restores clonogenic growth under oxidative stress. Cells were transfected with FASN cDNA or empty vector for 24 h, then treated with CoCl_2_ (16 h) or H_2_O_2_ (30 min), as indicated. Immunoblotting confirms FASN expression under the H_2_O_2_ condition. The data are representative of 2 independent experiments. Data are presented as mean ± SEM for each group and were analyzed by Student’s *t* test (**A**, **F**, **G**). ^*^P < 0.05; ^**^P < 0.01; ^***^P < 0.001; ^****^P < 0.0001.

To determine whether HIF-1α contributes to antioxidant pathway activity in CRC cells, we introduced HRE, SRE, and ARE transcriptional reporters into HIF-1α wild-type (sgRNA control) and HIF-1α knockout HCT116 cells. Reporter readouts were markedly reduced in HIF-1α knockout cells, with the strongest suppression observed for the HRE reporter and a clear decrease also seen for SRE and ARE activity (Fig. 6A), indicating that HIF-1α is required to sustain these stress-response transcriptional states in HCT116 cells. Because ARE activity is regulated by NRF2, we next examined NRF2 protein levels. Compared with controls, HIF-1α-KO cells exhibited substantially reduced NRF2 under basal conditions and after stimulation with IGF1 or insulin, despite compensatory induction of HIF-2α (Fig. 6B). Reduced NRF2 in HIF-1α-KO cells coincided with diminished pathway activation downstream of growth factors (Fig. 6B), consistent with impaired coupling between growth-factor signaling and stress-adaptive programs. Conversely, CRC lines with higher HIF-1α activity displayed higher NRF2 and FASN levels under basal and growth factor-stimulated conditions, supporting a relationship between HIF-1α status and NRF2 associated antioxidant capacity across CRC models (Fig. 6C). We next tested whether FASN contributes to NRF2 accumulation in HIF-1α high cells. In HRE-positive HCT116 cells, FASN knockdown reduced NRF2 protein levels at baseline and following oxidative stress challenge (H₂O₂) (Fig. 6D), suggesting that FASN is required to maintain NRF2 abundance under these conditions. Consistent with impaired oxidative stress buffering, FASN knockdown increased oxidative damage following H₂O₂ exposure, as indicated by elevated lipid peroxidation detected with ROS-665 (Fig. 6E). In the short-term 3-(4,5-dimethylthiazol-2-yl)-2,5-diphenyltetrazolium bromide (MTT) assay, knockdown of FASN or GLUT3 had minimal effects on viability under glucose deprivation (Glu(-), glycolysis inhibition (2DG), or lactic acid exposure, whereas viability was more strongly reduced under hypoxia-mimicking (CoCl_2_) and oxidative (H_2_O_2_) stress (Fig. 6F). In this same assay, GLUT1 knockdown also decreased viability under CoCl_2_, consistent with a contribution of glucose transport to hypoxia adaptation (Fig. 6F). Finally, to determine whether HIF-1α is required for these stress-resistant phenotypes, we assessed viability of HIF-1α-KO clones under stress. Compared with controls, HIF-1α-KO cells showed reduced capacity to withstand both hypoxia-mimicking and oxidative stress challenges (Fig. 6G), indicating that HIF-1α is upstream of the integrated stress-adaptation program. Restoration experiments further supported the role of FASN as an effector of this state, as FASN rescue increased clonogenic survival of FASN-KO cells under oxidative stress conditions (Fig. 6H). Collectively, these results indicate that HIF-1α sustains NRF2-associated antioxidant pathway activity and that downstream metabolic nodes, particularly FASN and GLUT3, confer complementary protection against oxidative and hypoxic stress to support CRC cell fitness.

### Targeting HIF-1α with LNP formulated echinomycin suppresses intratumoral HIF-1α, SREBP1, and NRF2 programs and inhibits CRC growth and metastasis

**Fig. 7:**
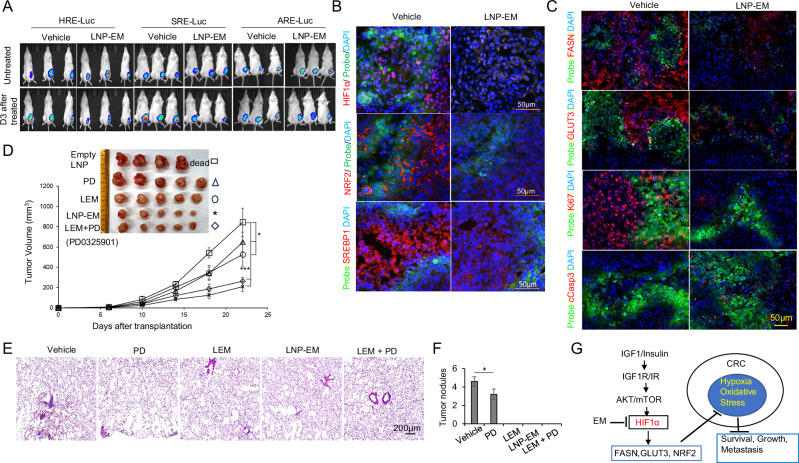
Targeting HIF-1α with LNP-Echinomycin suppresses intratumoral hypoxia/oxidative-stress programs and blocks CRC growth and lung metastasis. **A** HRE+ HCT116 cells stably expressing luciferase reporters (HRE-Luc, SRE-Luc, or ARE-Luc) were implanted in the inguinal area of NSG mice. Representative bioluminescence images are shown before treatment and 3 days after a single intravenous dose of LNP-Echinomycin (LNP-EM) (250 µg/kg) or vehicle. **B** Three days after treatment, mice received an intraperitoneal hypoxia probe (16 h), tumors were harvested, cryosectioned, and stained to visualize intratumoral hypoxia/oxidative-stress signaling, including HIF-1α and NRF2 in relation to probe-positive regions (DAPI counterstain). Representative images are shown. **C** Serial tumor sections stained to assess spatial expression of FASN and GLUT3 relative to probe-positive regions and tumor cell fate markers (Ki67 for proliferation; cleaved Caspase-3 for apoptosis). Representative images comparing vehicle vs LNP-EM are shown. **D** Primary tumor growth kinetics following treatment with empty LNP (vehicle control), PD0325901 (PD), liposomal echinomycin (LEM), LNP-EM, or LEM+PD; representative endpoint tumors are shown. Mean tumor volumes ± SEM are plotted on the *y* axis for each group (*n* = 5 per group) and were analyzed by two-way ANOVA. **E** Representative H&E images of lung sections showing metastatic lesions across treatment groups. **F** Quantification of lung metastatic burden (tumor nodules) from 10 fields per mouse using FFPE sections; *n* = 5 mice/group (statistics as indicated). **G** Working model: IGF1/insulin-IGF1R/IR-AKT/mTOR signaling and intratumoral hypoxia stabilize HIF-1α, which promotes CRC survival, growth, and metastasis via induction of FASN/GLUT3 and antioxidant/hypoxia-tolerance programs; echinomycin (EM) blocks HIF-1α-driven adaptation. The data are representative of 2 independent experiments.

To test whether pharmacologic inhibition of HIF-1α transcriptional activity can disrupt the stress-adaptive tumor state in vivo, we implanted HRE-positive HCT116 cells carrying luciferase reporters driven by HRE, SRE, or ARE elements into the inguinal region of NSG mice. Robust reporter signals were detected in established tumors, and intravenous administration of lipid nanoparticle (LNP)-formulated echinomycin (LNP-EM) produced a rapid reduction in HRE, SRE, and ARE reporter activity within 3 days compared with vehicle treatment (Fig. 7A), indicating suppression of hypoxia, lipogenic, and antioxidant transcriptional programs in tumors. We next mapped pathway activity within tumor microregions using a hypoxia probe to define hypoxic and peri-hypoxic zones. Immunofluorescence analysis demonstrated induction of HIF-1α and NRF2 in tumor areas associated with hypoxia probe signal, and LNP-EM markedly reduced both HIF-1α and NRF2 staining (Fig. 7B). In contrast, SREBP1 signal was most prominent in tumor regions beyond the hypoxic core (Fig. 7B), consistent with activation of anabolic programs in better-oxygenated, proliferative compartments. Consistent with this spatial organization, the HIF-1α downstream targets FASN and GLUT3 were enriched in regions outside the most hypoxic zones and overlapped with Ki67 positive proliferating tumor cells (Fig. 7C). LNP-EM treatment suppressed induction of FASN and GLUT3, reduced Ki67 staining, and increased cleaved caspase 3 positivity, supporting reduced proliferation and enhanced apoptosis following HIF-1α pathway inhibition (Fig. 7C). We then evaluated the therapeutic impact of HIF-1α inhibition on tumor growth and metastasis. LNP-EM significantly inhibited primary tumor growth compared with vehicle and outperformed liposomal echinomycin (LEM) and the MEK inhibitor PD0325901 administered alone under the tested conditions (Fig. 7D). Histological analysis of lungs demonstrated that echinomycin formulations, particularly LNP-EM, strongly reduced lung metastatic burden, whereas PD0325901 alone produced only modest effects (Fig. 7E, F). Combination treatment with LEM and PD0325901 augmented inhibition of primary tumor growth and lung metastasis relative to single agent controls (Fig. 7D–F), consistent with partial complementarity between MAPK pathway blockade and inhibition of stress-adaptive transcriptional programs. Together, these data support a model in which rapid tumor expansion generates hypoxia and oxidative stress that activate HIF-1α and NRF2 programs and promote outgrowth of proliferative tumor cells beyond hypoxic zones through induction of metabolic effectors, including FASN and GLUT3. Pharmacologic inhibition of HIF-1α DNA binding with echinomycin, particularly when delivered by LNP formulation, suppresses these intratumoral stress-response programs, decreases proliferation, increases apoptosis, and limits tumor growth and metastatic dissemination (Fig. 7G).

## Discussion

Rapid tumor proliferation is frequently accompanied by intratumoral hypoxia and elevated oxidative stress, creating strong selective pressure for metabolic programs that support both bioenergetics and stress adaptation. In this study, we combined functional reporter readouts with genetic perturbation and in vivo validation to define how HIF-1α-driven transcriptional programs shape colorectal cancer (CRC) growth and metastasis. Our data support a model in which a HIF-1α-high subpopulation (HRE+) is enriched for metastatic competence and stress tolerance. Notably, the most aggressive phenotypes emerged when hypoxic signaling and growth-factor signaling converged, enabling HIF-1α to coordinately promote glucose uptake, lipid biosynthesis, and antioxidant defense.

A key mechanistic insight from our work is that HIF-1α directly engages the human FASN promoter and activates FASN transcription, even in the absence of canonical SREBP1 activation. This provides a molecular explanation for how HIF-1α-high CRC cells sustain robust lipogenic output and suggests that hypoxic signaling can, in specific contexts, bypass the classical SREBP1-centric model of FASN regulation. SREBP1 remains an established master regulator of lipogenic enzyme genes, and extensive prior work has defined direct SREBP target genes and delineated nutritional and insulin-driven activation of the SREBP axis [37–40]. Our findings extend this framework by positioning HIF-1α as an additional, context-dependent driver of FASN expression, particularly in settings where hypoxia and oxidative stress are prominent.

Functionally, elevated FASN and GLUT3 appear to act as complementary downstream effectors of HIF-1α that promote survival under metabolic stress. GLUT3 supports high-capacity glucose uptake that can fuel both glycolysis and mitochondrial metabolism, consistent with the emerging view that many tumors deploy mixed metabolic strategies rather than a strict glycolysis-only state. In parallel, FASN-driven lipid synthesis can reinforce membrane integrity, expand signaling-lipid availability, and support redox buffering. In our system, FASN upregulation was tightly linked to NRF2 induction and reduced oxidative injury, supporting the concept that lipogenesis can couple to antioxidant defense programs. This aligns with reports that NRF2 blockade suppresses colon tumor angiogenesis by disrupting hypoxia-driven HIF-1α signaling [41], and that HIF-1α can maintain redox homeostasis through stress-adaptive bioenergetic programs, including glutamine-dependent pathways [42]. More broadly, resistance to ferroptosis has been increasingly connected to HIF-1α-regulated metabolic rewiring, including mechanisms that promote lactate production and activate amino-acid transport and antioxidant pathways [43].

Epidemiologic and clinical literature also support links between diabetes and CRC risk and outcomes [16]. Given that insulin and IGF1 converge on AKT and mTOR to promote HIF-1α accumulation in our system, our findings provide a biologically plausible framework in which hyperinsulinemic states reinforce a HIF-1α-dependent stress-adaptive program. This may help explain reported associations between diabetes and adverse CRC outcomes [14–16].

Finally, our results connect with emerging work linking GLUT3-associated programs to metastatic progression and survival. A Glut3–YAP-dependent circuit has been reported to rewire tumor metabolism and promote metastatic progression in CRC [31], and YAP-driven suppression of autophagy via Bcl-2 upregulation has also been implicated in CRC progression [44]. While our study centers on HIF-1α as the upstream coordinator of GLUT3 and FASN in stress-adapted states, our data are consistent with a broader model in which HIF-1α interfaces with multiple transcriptional hubs, including SREBP1 and NRF2, to integrate nutrient acquisition, survival signaling, and oxidative-stress control.

In summary, our study supports a model in which HIF-1α drives a coupled glucose–lipid–redox program through GLUT3 and FASN to promote CRC cell survival, growth, and metastasis under hypoxic and oxidative stress. By defining HRE- and SRE-based functional states and demonstrating direct HIF-1α control of FASN transcription, we provide a mechanistic rationale for targeting the HIF-1α–FASN/GLUT3 axis, including with nanoformulated HIF-1α inhibitors, as a strategy to blunt stress-adaptive metabolic fitness in aggressive CRC.

## Supplementary information

supplemental figures

## Data Availability

The datasets generated and/or analyzed during the current study are not publicly available but are available from the corresponding author upon reasonable request. All data supporting the conclusions of this article are included within the article and its supplementary files.
