# Supplementary material for: HIF-1α integrates lipogenic FASN and glycolytic GLUT3 to overcome intratumor oxidative and hypoxic stress for colorectal cancer metastasis
Source: Oncogene. 2026 Jun 3;45(27):2671–82. doi: 10.1038/s41388-026-03738-4 (PMC13314538; doi:10.1038/s41388-026-03738-4)
Supplement: Supplementary file 1 — supplemental figures [file 41388_2026_3738_MOESM1_ESM.pdf]

Supplemental Figures

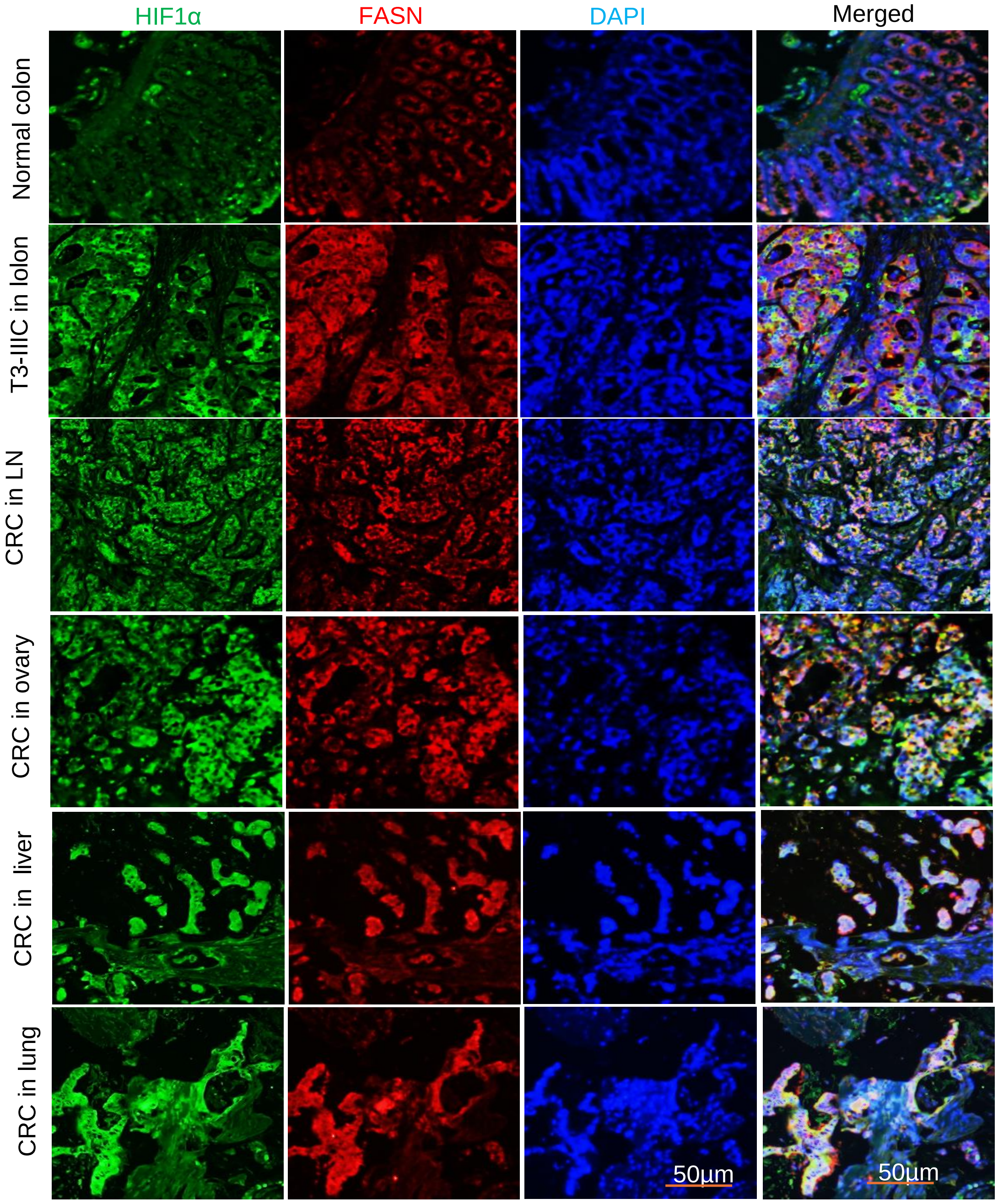

Fig.S1 Co-expression of HIF1α and FASN in metastatic CRC tissues.
Tissue microarray slide was stained with primary antibodies of HIF1α and FASN, then Alexa Fluor-conjugated secondary antibodies after washing.

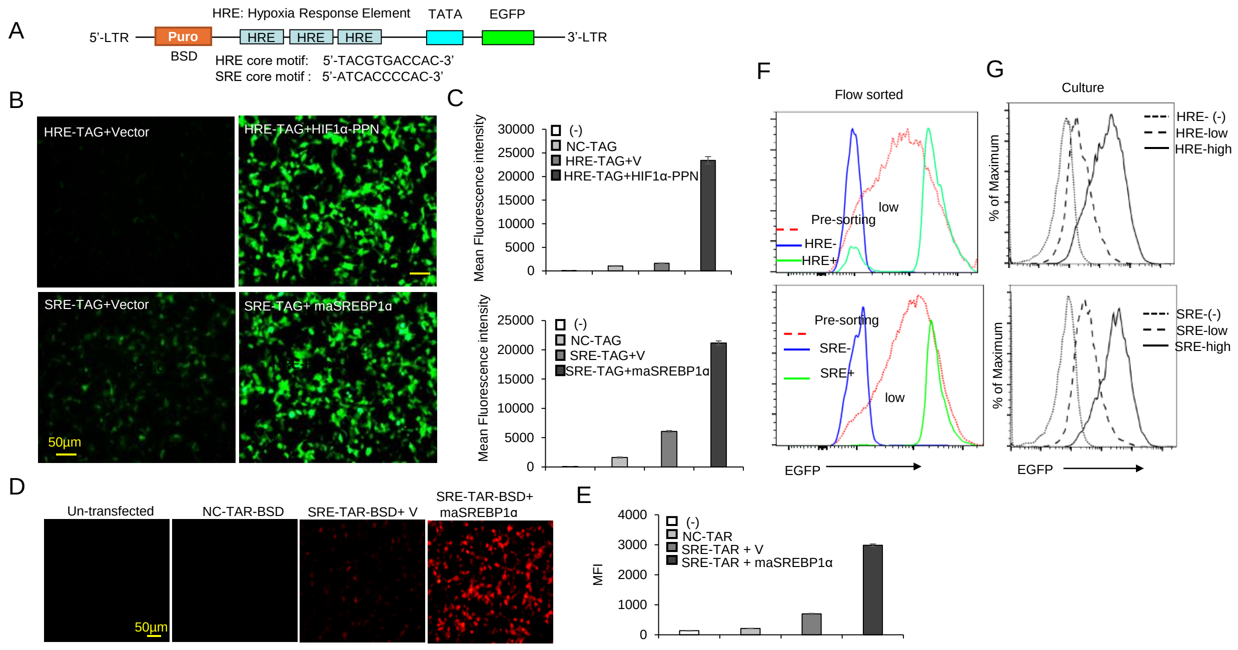

Fig.S2 Reporter specificity and FACS results of HRE and SRE. A. Map for HRE and SRE reporters. **B-F.** HEK293FT cells were transiently transfected with these reporters and cDNA plasmids as indicated for 32 hours (B,C) or 16 hours (D,F) before taking pictures and quantitation by flowcytometry. **G,H.** HCT116 cells were transduced lentivirus of HRE or SRE reporter to get puromycin-resistant stable cells before performing fluorescence-activated cell sorting (FACS) and culture of sorted cells.
